# Canine Tooth Position and Its Relationship with the Perpendicular Line to the Nasal Ala in an Iranian Population: Relationship of Canine with the Perpendicular Line to the Nasal Ala

**DOI:** 10.31661/gmj.v13iSP1.3568

**Published:** 2024-12-08

**Authors:** Zeinab Zahedi, Sayed Shojaedin Shayegh, Yasaman Sherafatmand, Mohammad Amin Bafandeh

**Affiliations:** ^1^ Faculty of Dentistry, Shahed University, Tehran, Iran; ^2^ Department of Prosthodontics, Faculty of Dentistry, Shahed University, Tehran, Iran; ^3^ Department of Dental Biomaterials, School of Dentistry, Tehran University of Medical Sciences, Tehran, Iran

**Keywords:** Anatomic Landmarks, Cuspid, Esthetics, Dental, Nasal Cartilages

## Abstract

**Background:**

This study aimed to assess the canine tooth position and its relationship with the line drawn perpendicular to the nasal ala in an Iranian population.

**Materials and Methods:**

This analytical epidemiological field study was conducted on 100 males and 100 females presenting to the dental clinic of Shahed Dental School in June 2023. Digital photographs were obtained from the patients under standardized conditions. Reference lines were drawn perpendicular to the nasal alae and continued down to the lower lip (AI lines). The distance from the canine cusp tip (AICT) and distal contact point of the canine tooth (AICD) to the AI line was measured bilaterally using Digimizer software version 6. Data were analyzed by t-test (alpha=0.05).

**Results:**

The AICT and AICD were significantly larger than zero in males and females (P0.05). The mean AICT and AICD in males were more significant than in females by 2-2.5 mm. No significant difference was found in the right and left canine positions, neither in males nor in females (P0.05).

**Conclusion:**

According to the results, the AI line and gender are both effective in determining the canine position in the study population and, therefore, should be considered in designing an esthetically pleasant denture. The canine position determined adequately for one side may also be used for the other side since no significant difference was found in the right and left canine positions.

## Introduction

The positive role of an attractive face and a pleasant smile in personal and social life has been well confirmed [1]. Leveled and aligned teeth are prominent in smile attractiveness and facial beauty, and canine teeth are essential in dental esthetics and occlusion [2]. Canine teeth have a strategic position in the dental arch and are crucial in smile esthetics, masticatory function, occlusion, and supporting the facial muscles [3]. They resemble incisors from the proximal view and premolars from the labial view [2]. In the case of canine tooth loss, its prosthetic replacement with a dental bridge would be difficult due to its position [4]. Thus, determining the vertical dimension of occlusion (VDO) is an essential step in prosthetic rehabilitation of dental arch [5]. Incorrect VDO can lead to several orofacial problems, such as bruxism, muscle pain, and temporomandibular disorders. Also, increased VDO can adversely affect facial esthetics [6]. Thus, prosthodontists often prefer a shorter VDO.

Several methods are available to determine VDO. The mechanical methods include anthropometric measurements, cephalometric analysis, facial profile assessment, and facial landmarks measurement [6]. However, comprehensive information for precise determination of the shape, size, and harmony of anterior teeth and their relationship with facial landmarks needs to be improved, and the available data in this regard are controversial [7].

Racial and gender disparities in the average measurements of anterior dentition have been documented [8]. Nonetheless, the findings presently accessible are exclusively relevant to particular demographic groups, underscoring the imperative for targeted investigations within various populations [9].

The main parameters involved in the esthetic appearance and harmony of the maxillary anterior teeth include the size, shape, leveling and alignment, and golden ratio of maxillary anterior teeth, especially central incisors, from the frontal view [5]. Also, the morphology of the anterior teeth should be in harmony with the facial morphology to maximize facial esthetics.

The selection of anterior teeth is an essential step in fabricating complete dentures. There needs to be a consensus regarding the best method of artificial tooth selection for dentures. This process is incredibly complicated when there is no record of the natural tooth dimensions of patients [10]. According to the literature, anatomical measurements are extensively used to aid in determination of proper size of maxillary anterior teeth, especially canine, for an esthetically ideal and functional denture, such as the interzygomatic width, intercommissural width, interalar width, interpupillary distance, and intercanthal distance [8][11][12].

Considering the reported differences in the mean tooth size in different racial and ethnic groups and males and females [8], this study aimed to assess the canine tooth position and its relationship with the line drawn perpendicular to the nasal ala in an Iranian population.

## Materials and Methods

**Table:** Table1. Mean AICT and AICD in Males and Females on the Right and Left Sides (n=100; Degree of Freedom=17)

**Gender**	**Variables**	**Statistic**	**Mean**	**P-value**	**Mean difference**	**95** **%** **Confidence Interval of the Difference**	
						**Minimum**	**Maximum**
	AICD-Left	11.28	1.12 ± 4.01	0.000	3.674	2.148	6.875
**Female**	AICD- Right	12.02	1.07 ± 3.03	0.000	3.259	1.453	4.859
	AICT-Left	11.98	0.98 ± 99/	0.000	4.687	2.657	4.985
	AICT-Right	12.96	1.18 ± 4.12	0.000	4.445	2.425	6.958
	AICD-Left	17.59	2.15 ± 7.02	0.000	5.425	3.369	7.850
**Male**	AICD- Right	16.54	0.67 ± 6.98	0.000	5.026	3.065	6.985
	AICT-Left	18.02	1.21 ± 5.32	0.000	6.580	4.120	6.349
	AICT-Right	17.19	2.01 ± 22.	0.000	6.951	4.518	7.002

This analytical epidemiological field study was conducted on 100 males and 100 females presenting to the dental clinic of Shahed Dental School in June 2023. The study protocol was approved by the university’s ethics committee (IR.SHAHED.REC.1402.052).

### Eligibility Criteria

The inclusion criteria were age>18 years, Iranian ethnicity, having all six maxillary anterior teeth, absence of cracks, prosthetic crowns, proximal restoration, crowding, rotation, or disharmony in the maxillary anterior teeth, and no history of cosmetic procedures in the anterior maxilla or rhinoplasty. The exclusion criteria encompassed individuals with a documented history of orthodontic interventions or orthognathic surgical procedures, as well as those presenting with dental trauma, dental anomalies, or congenitally absent teeth, alongside patients who exhibited a history of asymmetry or congenital or developmental abnormalities.Eligible patients were selected by convenience sampling.

### Sample Size

The required sample size was calculated to be 200 patients using G-Power version 3.1.9.7 assuming alpha=5%, study power of 80%, and effect size (d) of 0.57.

### Photography

Informed consent was obtained from all patients, ensuring the confidentiality of their information. They were informed that their facial photographs would be used solely for research. Full-face frontal-view photographs were obtained from the patients using a D3500 digital camera (Nikon, Japan) with a 55-18 mm lens. For this purpose, the patients were seated on a chair against the wall such that the distance between the nasal tip of each patient and the background wall was 25 cm, and the distance between the camera and the wall was 1 m. The tripod location was marked on the floor, and its height was adjusted according to the patient's height so that the head was at the center of the field of view. One expert grapher obtained all photographs according to the standards. The background wall was covered with green cardboard measuring 70 x 50 cm to improve the image quality and standardize the frame of all photographs. The patient's head was in the natural head position and at the center of the field of view during photography. For this purpose, the patients were asked to look at the camera's center. The photographer made the necessary changes in the head position of patients.

Accordingly, a smiling digital photograph was obtained from each patient so that the canine cusp tip and the canine's distal surface were visible. All photographs were obtained under standardized conditions in a sufficiently lit room with no magnification and a fixed tripod and at a fixed distance. After photography, the photographers standardized the photographs by using the interpupillary line parallel to the horizontal plane (to ensure that the drawn lines are perpendicular to the nasal alae). Photoshop software (adobe,california,usa) was then used to standardize the images in color and light. The photographs were subsequently cropped using the green cardboard in the background. The size of the green cardboard was used to standardize the photographs.

### Landmark Identification

All images were uploaded in Digimizer version 6 (MedCalc software Ltd, Belgium). The dimensions of the green cardboard in the background were used to calibrate the software's digital ruler. The measurements were made in millimeters (mm). Reference lines were drawn perpendicular to the nasal alae and continued down to the lower lip (AI). The distance from the canine cusp tip (AICT) and distal contact point of the canine tooth (AICD) to the AI line was measured bilaterally. The measurements were made twice by two independent operators. Also, 10% of the images were randomly selected and measured again.

### Statistical Analysis

Data were analyzed using SPSS version 26 (SPSS Inc., IL, USA). The normality of data distribution was evaluated and confirmed by the Kolmogorov-Smirnov test (P>0.05). Thus, comparisons were made by one-sample t-test, independent t-test (for comparing males and females), and paired t-test (for comparing right and left sides) at a 0.05 significance level.

## Results

Table-1 presents the mean AICT and AICD in males and females on the right and left sides. The AICT and AICD were more than zero in males and females. The mean AICT and AICD in males were significantly more prominent than in females by 2-2.5 mm (P=0.00 for all).

A comparison of the values on the right and left sides of the face (Table-2) revealed no significant difference between the right and left sides, either in males or in females (P>0.05).

## Discussion

**Table:** Table2. Comparison of Right and Left AICD and AICT in Males and Females

			Paired Differences		
Gender			Mean	Std. Deviation	Std. Error Mean	95% Confidence Interval of the Difference		t	df	Sig. (2-tailed)
					Upper	Lower			
female	Pair 1		.115680	3.526977	.265894	-.452896	.458276	-.099	17	.553
	Pair 2	AICT-Left AICT-Right	-.625127	3.259874	.236548	-.325986	.625147	.415	17	.409
male	Pair 1	AICD-Left AICD- Right	-.452810	3.265849	.369521	-1.154203	0.124589	-1.223	17	0.115
	Pair 2	AICT-Left AICT-Right	-.628420	3.215789	.236584	-1.264755	.415897	-.702	17	.411

This study assessed the canine tooth position and its relationship with the line drawn perpendicular to the nasal ala in an Iranian population. The results showed that the AICT and AICD were significantly larger than zero in both males and females. The mean AICT and AICD in males were more significant than in females by 2-2.5 mm. No significant difference was found in the right and left canine positions, neither in males nor in females.

Most available studies on this topic focused on inter canine width or width of the anterior teeth. For instance, Khaneh Masjedi et al. [13] demonstrated that facial index significantly affected all the influential factors on smile attractiveness. Therefore, greater attention should be paid to facial index in treatment planning and mechanics to create a more attractive smile and improve patient satisfaction with the outcome. Nasiri et al. [14] reported a positive significant correlation between anterior tooth size and distance between the canine cusp tip and nasal ala. The mean size of facial and dental parameters was significantly different in males and females, and facial width, height, and maxillary central incisor width and height were greater in males than females. Their results agreed with the present findings regarding the more considerable distance between the canine cusp tip and the perpendicular line to the nasal ala in males than females. Hasanreisoglu et al. [10] reported a significant difference in central incisor and canine tooth dimensions based on gender, which agreed with the present results. Tarfa and Salih [15] measured the inter-canine and intermolar widths and vertical molar and canine distance and showed significant correlations between these parameters in both the maxilla and mandible. The maxillary inter-canine width and the maxillary and mandibular vertical molar distance had a moderate correlation.

In contrast, the correlation between the maxillary and mandibular molar vertical distance was highly significant. Pisulka et al. [16] measured the mesiodistal width of maxillary anterior teeth, intercanthal distance, interalar distance, and inter-commissural width. They showed a positive correlation between the inter-commissural width and mesiodistal width of maxillary anterior teeth. Srimaneekarn et al. [17] measured the nasal alar width and inter-canine distance. They used two reference lines of nasal alar line and inner canthal-alar distance to determine the canine position. They showed significant differences between males and females in all parameters.

Also, the nasal alar reference line yielded more favorable results than the inner canthal-nasal alar line; their results agreed with the present findings. Omrani et al. [8] discussed that dental clinicians may need to use the golden ratio as an index of treatment planning. The mean inter-commissural width to nasal width ratio and interzygomatic width to nasal width were greater in females than males [8]. Bonakdarchian and Ghorbanipour [18] showed significant correlations between the interalar width and inter canine width and the sum of widths of six maxillary anterior teeth in both males and females, which was in line with the present results regarding gender-related differences in facial parameters. In Thailand, Srimaneekarn et al. [17] used two reference lines of nasal alar and inner canthal-nasal alar lines to determine canine position. Consistent with the present results, all values significantly differed between males and females in their study. The mean AICD distance in their study was smaller than that in the present study in both males and females and on both the right and left sides, which may be due to racial differences. They showed that using the line drawn from the nasal ala perpendicular to the occlusal plane was more suitable than the AI line for the determination of the width of six anterior teeth in the Asian population because the A-line matches the distal contact point of the canine tooth in males while the AI line is always located at the distal of the canine tooth.

Nonetheless, the present results showed that although the AI line was always at the distal of the canine tooth, the canine position was significantly correlated with the AI line, and the AI line may be used to determine the canine position in the Iranian population. Accordingly, after drawing the AI line, the distal point of the canine can be identified at a 2 mm distance from it, and the canine cusp tip can be identified at a 4 mm distance from it in females. For males, 2 to 2.5 mm should be added to the abovementioned values.

The single-center design was the main limitation of this study. Future multi-center studies with a larger sample size must obtain more reliable and generalizable results. Also, the correlation of other facial landmarks with the position of teeth should be investigated in future studies.

## Conclusion

According to the results, the AI line and gender are both effective in determining the canine position in the study population and should be considered when designing an esthetically pleasant denture. The canine position determined adequately for one side can also be used for the other side since no significant difference was found in the right and left canine positions.

## Conflict of Interest

None declared.
